# Establishing reliable DNA barcoding primers for jumping plant lice (Psylloidea, Hemiptera)

**DOI:** 10.1186/s13104-023-06585-8

**Published:** 2023-11-08

**Authors:** Saskia Bastin, Diana M. Percy, Felipe Siverio

**Affiliations:** 1 Instituto Canario de Investigaciones Agrarias, Unidad de Protección Vegetal, C/ El Boquerón s/n, 38270 La Laguna, Tenerife Spain; 2 Universidad de La Laguna, Avda. Astrofísico Francisco Sánchez, SN. Edificio Calabaza-AN.2D Apdo. 456., 38200 La Laguna, Tenerife Spain; 3 Botany Department and Biodiversity Research Centre, University of British Columbia, Vancouver, BC Canada

**Keywords:** Psyllids, Molecular identification, Primer efficacy, Species identification, COI barcode, Standard barcode

## Abstract

**Objectives:**

DNA Barcoding has proven to be a reliable method for rapid insect identification. The success of this method is based on the amplification of a specific region, the ‘Folmer’ barcode region at the 5´ start of the cytochrome c oxidase 1 gene (cox1), with universal primers. Previous studies showed failures of standard “universal” primers to amplify this region in psyllids. The aim of the study was the design of a new alternative more reliable primer combination for taxa of the superfamily Psylloidea and its comparison with the performance of the standard “universal” Folmer-primers.

**Results:**

A newly designed degenerate forward primer LCOP-F was developed following comparison of the sequence alignment of the priming site of “universal” primer LCO1490 and the standard insect forward primer LepF1. When combined with the “universal” reverse primer, HCO2198, this new primer pairing was able to generate barcode sequence for all 36 species in 20 genera across the five families of psyllids tested in this study, and these primers were found to be more universally reliable across psyllid taxa than other primer pairs tested.

**Supplementary Information:**

The online version contains supplementary material available at 10.1186/s13104-023-06585-8.

## Introduction

Over 4000 species of psyllid, or jumping plant-lice, (Hemiptera: Psylloidea) are described worldwide [1] and it has been estimated that twice as many species are still undescribed [2]. Due to their host specific behaviour, some psyllids have been used as biological control agents of exotic plants [3, 4]. Others are among the most devastating pests worldwide due to their ability to transmit plant pathogens of the genus “*Candidatus* Liberibacter” [5], such as the tomato and potato psyllid (*Bactericera cockerelli* Šulc) associated with Zebra chip disease, and the Asian and African citrus psyllids (*Diaphorina citri* Kuwayama and *Trioza erytreae* Del Gercio, respectively) associated with huanglongbing disease [6–9]. Rapid and accurate identification of psyllid species is therefore crucial in many applications, including biosecurity and biodiversity assessments. However, morphological identification of psyllids is challenging for three main reasons: the lack of taxonomic keys, the lack of distinguishing morphological features in some immature stages and some adults (especially females in certain groups), and the presence of seasonal dimorphism [10].

Success of the DNA barcoding method, and construction of reference DNA barcode libraries for taxon identification, relies on the efficiency of “universal” primers to amplify the same gene region for many different taxa. The use of standard mitochondrial primers for invertebrate species identification and systematics was proposed by Folmer et al. [11] and Simon et al. [12], among others, with one particular gene, cytochrome c oxidase 1 gene (cox1), promoted as the best candidate region for a standard DNA barcode for animals [13]. In one of the earliest publications on DNA barcoding, Folmer et al. proposed the cox1 gene and a set of potentially “universal” primers (LCO1490/HCO2198) to amplify the 5’ end of cox1 across a diverse group of invertebrate phyla including molluscs, echinoderms, and tardigrades [11]. Subsequently, studies showed that these “universal” primers performed poorly for certain taxonomic groups [14–16].

The cox1 DNA barcode has proven to be a highly effective tool for identification in psyllids [10, 17, 18], but in most cases researchers use different sets of primers to amplify slightly different parts of cox1 and often need more than one primer set to obtain amplification across all focal taxa [10, 19]. The option to have a single primer pair that would reliably amplify and sequence across the superfamily Psylloidea as well as maximize the quantity and informativeness of the data has not been available for psyllids. Furthermore, the use of different primer pairs to amplify slightly different regions of cox1 prevents the construction of a comparative worldwide DNA barcode library for psyllids due to variable lengths and different placement of sequence alignments from different studies.

A screen of publicly available sequences of psyllid taxa in BOLD Systems and GenBank reveals that less than 30% of the species are represented by a cox1 sequence with length > 500 bp, which is a length proposed as a requirement for a standard barcode [20]. Furthermore, only 20% of these have been amplified with the “universal” primers proposed by Folmer et al. [11]. The remaining sequences (80%) have been amplified with many different primer pairs such as the modified primer set developed specifically for Lepidoptera [21] that became standard for many insect taxa, LepF1/LepR1 (but accounts for less than 5% of psyllid sequences); or various alternatives designed for specific geographically focused studies [19, 22–25] (see Additional file 1: Table S1).

**Fig. 1:**
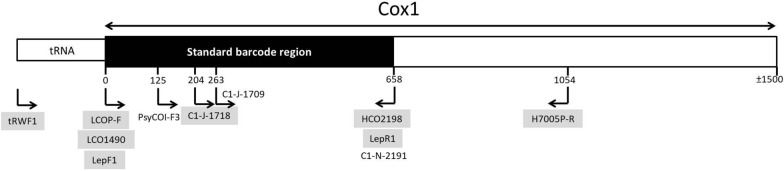
Schematic of the relative primer locations and expected sequence lengths of most of the primers used in published DNA Barcoding studies with insect and/or psyllid species. The primers used in this study are highlighted in grey

The poor amplification and universality of most of these primers positioned at the start of cox1 is a result of a not particularly conserved sequence in the first 100–200 bp of the gene. For this reason, a number of researchers have selected to use forward primers upstream of the start of the gene (e.g., primer pair C1-J-1718/C1-N-2191 from Simon et al. [12], C1-J-1709/HCO2198 used in Martoni et al. [17], and PsyCOI-F3 from Martoni et al. [19], (Fig. 1), but then yield a shorter total sequence length. We therefore focused on designing a new degenerate forward primer at the start of cox1 that can potentially be paired with a number of proven reverse primers. We tested the performance of this new forward primer on 36 species from 20 genera in five families of Psylloidea. In addition, we compared the efficacy of our new degenerate forward primer against previous standard primers, LCO1490/HCO2198 from Folmer et al. [11] and LepF1/LepR1 from Hebert et al. [21].

Our primary aim in introducing a new degenerate forward primer at the 5′ start of the cox1 gene is to help standardize DNA barcoding in psyllids and promote sequencing of the same primary psyllid barcode, even if in addition to other gene regions.

## Methods

### Species collection and DNA extraction

A total of 154 specimens from 36 species, in 20 genera across five of the seven families of Psylloidea as classified by Burckhardt et al. [26] were used in this study. Additional sample information is provided in Additional file 2: Table S2. All specimens were morphologically identified to species based on keys in Hodkinson [27], Hodkinson [28], Rapisarda [29] and Bastin et al. [30]. DNA was extracted from whole individuals using a Chelex non-destructive protocol [31] as follows: each specimen was immersed in 10 ml of Proteinase K (10 mg/ml) and 150 µl of Chelex diluted at 10% and incubated in the thermoblock at 55 °C for 12 h. The solution was used directly as the DNA extraction while the specimen was removed and retained as a DNA voucher, preserved in 70% ethanol and deposited in the Instituto Canario de Investigaciones Agrarias (ICIA), Spain.

### Primer design, PCR and sequencing

To design the new forward primer, sequences of cox1 and flanking regions from publicly available psyllid data (NCBI) (e.g., from complete mitochondrion) were assembled into an alignment using ClustalW in Mega 7 [32]. Additional sequences of the tRNA-W region upstream of the 5′ end of cox1, were also obtained using the primers tRWF1 [33] and LepR1 [21] (Additional file 2: Table S2). The tRWF1 primer located in the tRNA allowed us to sequence through the LCO primer binding site as the tRNA-W gene is located around 200 bp upstream of cox1 [33]. Altogether, we compared sequences from six of the seven recognized psyllid families (no samples or published sequences of Mastigimatidae were available) to design the new degenerate forward primer. Details of the samples and sources of the sequences used for the design of the forward primer are provided in Additional file 3: Table S3. Alignment of sequences was performed with ClustalW in Mega 7 [32], and then checked and adjusted manually before comparing base calls with published primer sequences. Based on variable sites across the primer length, we designed the new degenerate forward primer, LCOP-F (5′ AGAACWAAYCATAAAAYWATTGG-3′) as a modification of the universal forward primer LCO1490. In contrast, the priming site of the reverse primer, HCO2198, is relatively conserved among psyllid species (see Additional file 4: Fig. S1), and so we decided to pair our new degenerate forward primer with HCO2198, which gives an amplified length of ~ 660 bp.

We compared the formation of reproducible discrete bands after electrophoresis of PCR products of our newly designed primer LCOP-F coupled with HCO2198 with the “universal” primer pair LCO1490/HCO2198 as well as several other published primer combinations used for amplifying cox1 that focus on the 5′ end of the gene typically considered the standard barcode region for insects. Details of primer sequences, annealing temperatures and citations are provided in Table 1; Fig. 1 provides a schematic of the relative primer locations and expected sequence length. The same DNA extracts were used for the different primer pairs for direct comparison (Additional file 2: Table S2). PCR amplifications were then performed in a 25 µl final reaction volume containing 0.4 µM of each primer, 3 mM MgCl_2_, NH4 buffer (1X), 0.2 mM of each dNTP, 0.4 mg/ml of acetylated bovine serum albumin (BSA), 0.02 unit/µl of Taq-polymerase (Bioline) and 2 µl of DNA extract (concentration not determined). PCRs were carried out in a Swift™ Maxi Thermal Cyclers (ESCO Technologies) applying the following thermal step: initial denaturation for 4 min at 94 °C, followed by 39 cycles of 30 s at 94 °C, 30 s at annealing temperature of 50 °C and 45 s at 72 °C, then a final extension step of 10 min at 72 °C. PCR products were enzymatically cleaned in microtubes with 0.025 unit/µl rAPid alkaline phosphatase (Roche) and 50 unit/ml exonuclease I (BioLabs) for 15 min at 37 °C followed by 15 min at 85 °C. Then the purified products were sequenced in both directions at Macrogen Inc. (Madrid, Spain). Sequences were checked, edited and assembled for both directions with CLUSTALW within the MEGA 7 software [32].

**Table 1:** Details of primer sequences, amplicon length, annealing temperatures and references of the primers used to amplify the cytochrome oxidase 1 gene (cox1) in this study

Primer pair	References	Function	Sequence (5´–3´)	Tm (°C)	Amplicon lenght (bp)
C1-J-1718	Simon et al. [12]	Forward	GGAGGATTTGGAAATTGATTAGTTCC	50	850
H7005P-R	Percy and Cronk [36]	Reverse	TGAGCTACTACRTARTATGTRTCATG		
LCOP-F	From this study	Forward	AGAACWAAYCATAAAAYWATTGG	48	658
HCO2198	Folmer et al. [11]	Reverse	TAAACTTCAGGGTGACCAAAAAATCA		
LepF1	Hebert et al. [13]	Forward	ATTCAACCAATCATAAAGATATTGG	50	658
LepR1	Hebert et al. [13]	Reverse	TAAACTTCTGGATGTCCAAAAAATCA		
tRWF1	Park et al. [14]	Forward	AACTAATARCCTTCAAAG	50	± 860
LepR1	Hebert et al. [13]	Reverse	TAAACTTCTGGATGTCCAAAAAATCA		
LCO1490	Folmer et al. [11]	Forward	GGTCAACAAATCATAAAGATATTGG	48	658
HCO2198	Folmer et al. [11]	Reverse	TAAACTTCAGGGTGACCAAAAAATCA		

## Results and discussion

**Fig. 2:**
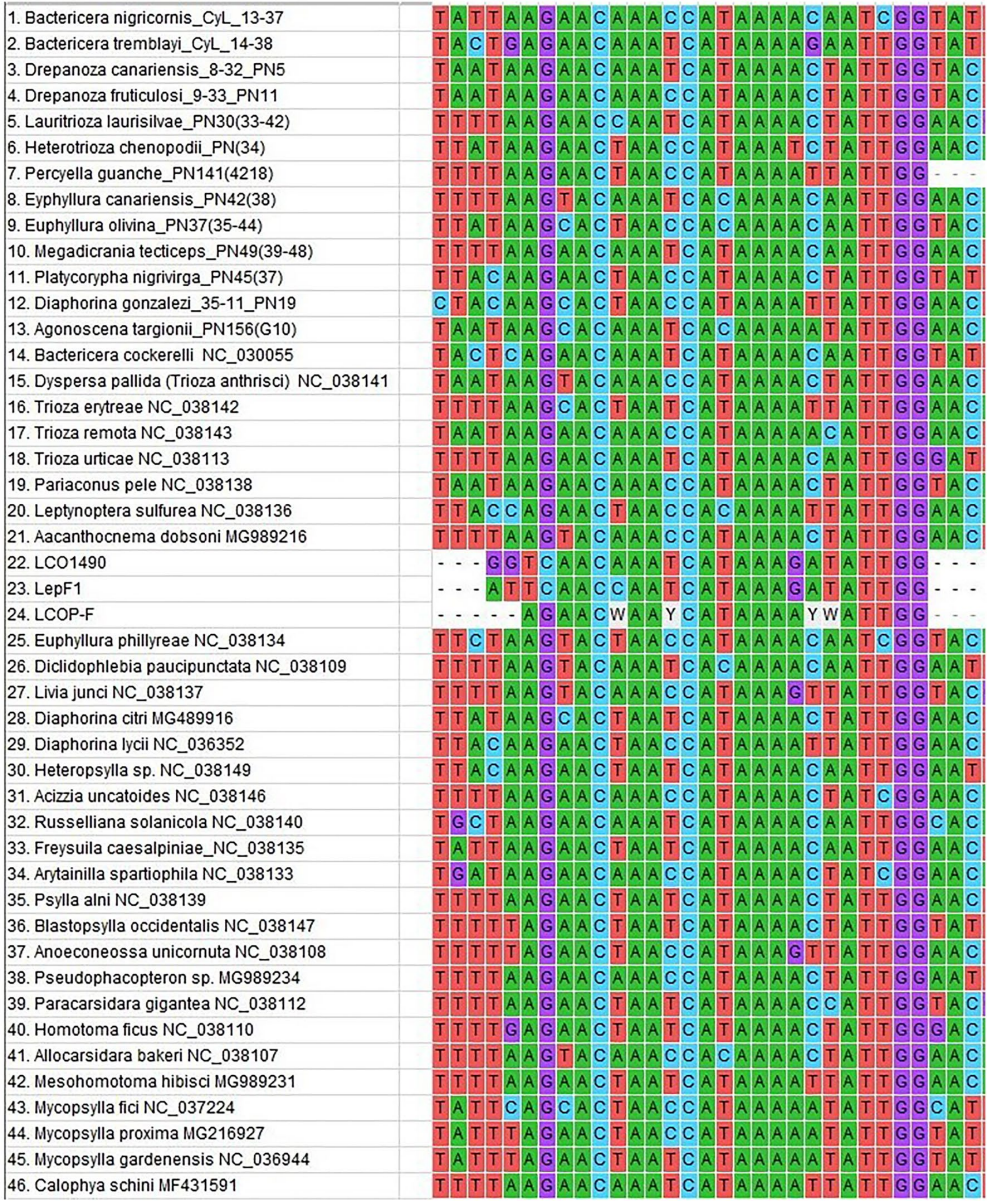
Snap shot of the multiple alignment of the forward primer binding site of the psyllid cox1 sequences obtained from GenBank and during this study including also the sequence of the “universal” primer LCO1490, the standard insect forward primer LepF1 and our new degenerate primer LCOP-F.

PCR amplification products were obtained with the new degenerate forward primer, LCOP-F, coupled with reverse primer HCO2198 from Folmer et al. [11] for 146 of the 154 specimens, resulting in a 95% success rate. This primer combination amplified the first 658 bp of cox1 for all 36 species tested, which represents five of the seven recognized families [26]. Sequences have been deposited in GenBank database (Accession numbers: OR027185-OR027257, OR029451-OR029453). None of these sequences showed frame-shifts or stop-codon. The result of a primer efficiency comparison is shown in Table 2, and Additional file 2: Table S2 and Additional file 5: Table S5, respectively. As expected, the lowest amplification success was obtained with the insect barcode primer pairs: LepF1 with LepR1 and LCO1490 with HCO2198, with only 13% and 35% of the species successfully amplified respectively. According to Hajibabaei et al. [34], new primer design should be considered if less than 95% success rate is obtained with existing primers for a broad range of species in the target group. Alignment of our sequences with 31 species obtained from GenBank revealed nucleotide mismatches with two of the commonly used barcoding forward primers, LCO1490 and LepF1 (see Fig. 2), that likely explains the failure of these universal primers to generate PCR amplicons for most psyllid species tested. This is consistent with recent studies that have revealed high sequence variability in the primer site of the Folmer forward primer LCO1490 [33, 35]. The primer pair, tRWF1/LepR1 successfully amplified 78% of species tested (47 of the 60 specimens), while the pair C1-J-1718/H7005P-R (the latter a degenerate reverse primer from Percy & Cronk [36]) successfully amplified all but one species tested (119 of the 143 specimens tested, 83%). However, although showing a good success rate, this latter primer pair avoids the variable 5′ end of cox1 and therefore does not recover the start of cox1 which is deemed important for a standard barcode region (Fig. 1). Our new degenerate forward primer, LCOP-F paired with HCO2198 gives an amplified length of ~ 660 bp, but it should be possible to extend this sequence length by pairing LCOP-F with other proven reverse primers (e.g., H7005P-R). Combining the two degenerate primers LCOP-F/H7005P-R provides a sequence length of ~ 1000 bp that could provide additional information for phylogenetical studies, but the efficacy of this primer combination has not been extensively tested in this study.

**Table 2:** Results of primer efficacy comparison with the number of specimens, species, genera and families tested and the number and percentage of species and specimens amplified

	Specimens tested	Species tested	Genera tested	Families tested	Species amplified	Species amplified (%)	Specimens amplified	Specimens amplified (%)
LCOP-F/HCO2198	154	36	20	5	36	100	146	95
C1-J-1718/H7005P-R	143	36	20	5	35	97	119	83
LCO1490/HCO2198	54	26	18	5	9	35	24	44
LepF1/LepR1	27	24	18	5	3	13	3	11
tRWF1/LepR1	60	25	16	4	18	72	47	78

Although our sampling includes only one member of family Carsidaridae, and the two smallest families are unrepresented (Calophyidae and Mastigimatidae), our sampled dataset spans the maximum phylogenetic distance within the Psylloidea, with the unsampled families phylogenetically nested among those families sampled. For many other Hemiptera groups (e.g., Auchenorrhyncha and Heteroptera) the LCO1490 forward primer sequence provides a 100% match, but from a survey of GenBank sequences some taxa in other Sternorrhyncha groups (e.g., Aphidomorpha, Aleyrodoidea, Coccoidea) and some of the wider hemipteroid groups (e.g., Psocoptera and Thysanoptera) there are similar mismatches at the primer binding site to those found in Psylloidea and therefore the new LCOP-F primer may also prove effective in these groups.

## Conclusion

The new degenerate primer coupled with one of the original “universal” primers successfully amplified the cox1 DNA barcode region for all species tested representing five of the seven recognised families in Psylloidea. In addition, these primers were able to successfully sequence the DNA barcode for all species tested. This study also confirmed that the forward priming site at the start of cox1 is not particularly conserved in psyllids, which likely explains the failure of standard barcode primers to amplify this 5’ region of cox1. Finally, the new degenerate forward primer will improve the implementation of DNA barcoding in psyllids by providing a standard option, and will also facilitate the establishment of a DNA barcode library for rapid and accurate identification of psyllid species and more effective comparison of sequence data from different studies.

## Limitations

Although the new degenerate forward primer has been tested on a phylogenetically broad representation of psyllids, further work is needed to confirm its efficacy in a larger sample of psyllid species, as well as in some of the tropical groups not or only poorly represented in this study (e.g., in families Calophyidae, Carsidaridae and Mastigimatidae), and also its potential utility for other Hemiptera and a wider range of insects.

### Supplementary Information

**Additional file 1: Table S1. **Publicly available sequences of psyllid taxa present in BOLD Systems with barcode length, primers used, length of the DNA barcode sequences > 500bp (Assessed on 19.01.2023).

**Additional file 2: Table S2. **Information of the samples used in this study with the results for each primer pair. Results: blank = non-tested; yes/no = formation or not of reproducible discrete bands after electrophoresis of PCR amplifications

**Additional file 3: Table S3. **Details of the samples and sequences used for the design of the forward primer.

**Additional file 4: Fig. S1. **Snap shot of the multiple alignment of the reverse primer binding site of the psyllid cox 1 sequences obtained from GenBank and during this study including also the sequence of the "universal" primer HCO2198 and the standard insect reverse primer LepR1.

**Additional file 5: Table S4. **Results of PCR amplifications of the 36 psyllid species analysed (20 genera and 5 families) with the different pairs of primers tested. X = no amplification; Yes/No = formation or not of reproducible discrete bands after electrophoresis of PCR amplifications

**Additional file 6. References.** Bibliographic references of supplementary information.

## Data Availability

Sequences generated for this study are deposited in GenBank: Accession numbers OR027185-OR027257 and OR029451-29453. All other data generated or analysed during this study are included in this published article and its additional files.

## Abbreviations

BOLD: Barcode of Life Data System
PCR: Polymerase chain reaction
