# Supplementary figures and images for: Establishing reliable DNA barcoding primers for jumping plant lice (Psylloidea, Hemiptera)
Source: BMC Res Notes. 2023 Nov 8;16:322. doi: 10.1186/s13104-023-06585-8 (PMC10634070; doi:10.1186/s13104-023-06585-8)

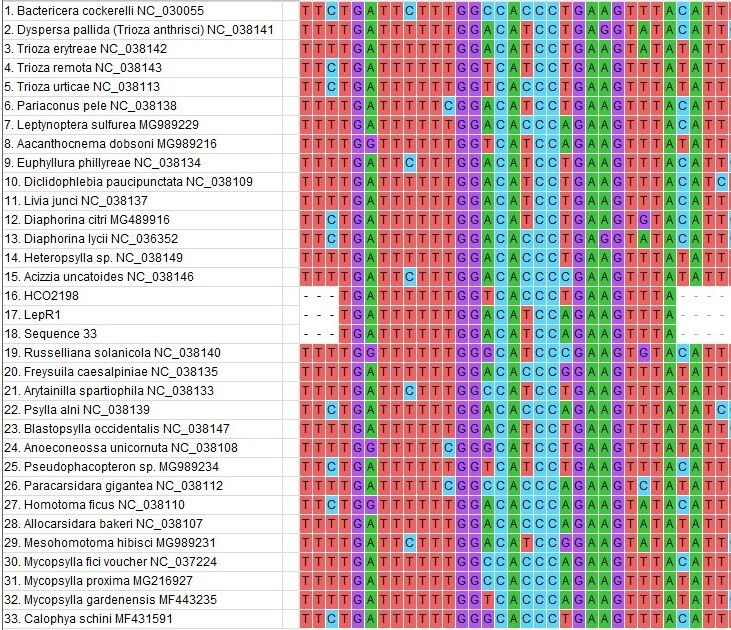

Supplement: Supplementary file 4 — Additional file 4: Fig. S1. Snap shot of the multiple alignment of the reverse primer binding site of the psyllid cox 1 sequences obtained from GenBank and during this study including also the sequence of the "universal" primer HCO2198 and the standard insect reverse primer LepR1. [file 13104_2023_6585_MOESM4_ESM.jpg]
