# Supplementary material for: Establishing reliable DNA barcoding primers for jumping plant lice (Psylloidea, Hemiptera)
Source: BMC Res Notes. 2023 Nov 8;16:322. doi: 10.1186/s13104-023-06585-8 (PMC10634070; doi:10.1186/s13104-023-06585-8)
Supplement: Supplementary file 6 — Additional file 6. References. Bibliographic references of supplementary information. [file 13104_2023_6585_MOESM6_ESM.docx]

**References**

Antolínez CA, Moreno A, Ontiveros I, Pla S, Plaza M, Sanjuan S, Palomo JL, Sjölund MJ, Sumner-Kalkun JC, Arnsdorf YM, Jeffries CJ, Ouvrard D, Fereres A. Seasonal abundance of psyllid species on carrots and potato crops in Spain. Insects. 2019;10(9):287.

Batovska J, Piper AM, Valenzuela I, Cunningham JP, Blacket MJ. Developing a non-destructive metabarcoding protocol for detection of pest insects in bulk trap catches. Scientific Reports. 2021;11(1):7946.

Benhadi‐Marín J, Félix‐Oliveira D, Carvalho MDRP, Mendes JL, Baptista P, Pereira JA. Presence and distribution of the African citrus psyllid in São Tomé Island. Journal of Applied Entomology. 2022;146(9):1212-1216.

Diaz R, Dickey AM, Shatters RG, Manrique V, Vitorino MD, Overholt WA. New species diversity revealed from molecular and morphological characterization of gall-inducing *Calophya* spp. (Hemiptera: Calophyidae) from Brazilian peppertree. Florida Entomologist. 2015;98(2):776-779.

Dittrich-Schröder G, Garnas JR, Arriagada-Cares D, Ahumada R, Hurley BP, Lawson SA, Slippers B. Diversity and introduction history of *Glycaspis brimblecombei* reflects a history of bridgeheads and distinct invasions. Frontiers in Forests and Global Change. 2021;4:783603.

Hebert PD, Ratnasingham S, Zakharov EV, Telfer AC, Levesque-Beaudin V, Milton MA, Pedersen S, Jannetta P, DeWaard JR. Counting animal species with DNA barcodes: Canadian insects. Philosophical Transactions of the Royal Society B: Biological Sciences. 2016;371:20150333.

Ibrahim YE, Paredes-Montero JR, Al-Saleh MA, Widyawan A, He R, El Komy MH, Al Dhafer HM, Kitchen N, Gang DR, Brown JK. Characterization of the Asian Citrus Psyllid-‘*Candidatus* Liberibacter asiaticus’ Pathosystem in Saudi Arabia Reveals Two Predominant CLas Lineages and One Asian Citrus Psyllid Vector Haplotype. Microorganisms. 2022;10:1991.

Khamis FM, Rwomushana I, Ombura LO, Cook G, Mohamed SA, Tanga CM, Nderitu PW, Borgemeister C, Sétamou M, Grout TG, Ekesi S. DNA barcode reference library for the African citrus triozid, *Trioza erytreae* (Hemiptera: Triozidae): vector of African citrus greening. Journal of economic entomology. 2017;110(6):2637-2646.

Kuznetsova VG, Labina ES, Shapoval NA, Maryańska-Nadachowska ANNA, Lukhtanov, VA. *Cacopsylla fraudatrix* sp. n. (Hemiptera: Psylloidea) recognised from testis structure and mitochondrial gene COI. Zootaxa. 2012;3547(1):55-63.

Martoni F, Blacket MJ. Description of an Australian endemic species of *Trioza* (Hemiptera: Triozidae) pest of the endemic tea tree, *Melaleuca alternifolia* (Myrtaceae). Plos one. 2021;16(9):e0257031.

DeWaard JR, Ratnasingham S, Zakharov EV, Borisenko AV, Steinke D, Telfer AC, Perez KHJ, Sones JE, Young MR, Levesque-Beaudin V, Sobel CN, Abrahamyan A, Bessonov K, Blagoev G, deWaard SL, Ho C, Ivanova NV, Layton KKS, Lu L, Manjunath R, McKeown JTA, Milton MA, Miskie R, Monkhouse N, Naik S, Nikolova N, Pentinsaari M, Prosser SWJ, Radulovici AE, Steinke C, Warne CP, Hebert PD. A reference library for Canadian invertebrates with 1.5 million barcodes, voucher specimens, and DNA samples. Scientific data. 2019;6(1):308.

Nokkala S, Kuznetsova VG, Nokkala C. Characteristics of parthenogenesis in *Cacopsylla ledi* (Flor, 1861) (Hemiptera, Sternorryncha, Psylloidea): Cytological and molecular approaches. Comparative Cytogenetics. 2017;11(4):807.

Nokkala C, Kuznetsova VG, Rinne V, Nokkala S. Description of two new species of the genus *Cacopsylla* ossiannilsson, 1970 (Hemiptera, Psylloidea) from northern Fennoscandia recognized by morphology, cytogenetic characters and COI barcode sequence. Comparative Cytogenetics, 2019;13(4):367.

Nokkala C, Kuznetsova VG, Shapoval NA, Nokkala S. Phylogeography and *Wolbachia* Infections Reveal Postglacial Recolonization Routes of the Parthenogenetic Plant Louse *Cacopsylla myrtilli* (W. Wagner 1947), (Hemiptera, Psylloidea). Journal of Zoological Systematics and Evolutionary Research. 2022;2022:1-12.

Om N. The roles of psyllids, host plants and environment in the aetiology of huanglongbing in Bhutan (Doctoral dissertation, Western Sydney University (Australia). 2017.

Percy DM, Crampton-Platt A, Sveinsson S, Lemmon AR, Moriarty Lemmon E, Ouvrard D, Burckhardt D. Resolving the psyllid tree of life: phylogenomic analyses of the superfamily Psylloidea (Hemiptera). Systematic Entomology. 2018;43:762-776.

Rasowo BA, Copeland RS, Khamis FM, Aidoo OF, Ajene IJ, Mohamed SA, Sétamou M, Ekesi S, Borgemeister C. Diversity and phylogenetic analysis of endosymbionts from *Trioza erytreae* (Del Guercio) and its parasitoids in Kenya. Journal of Applied Entomology. 2021;145(1-2):104-116.

Rwomushana I, Khamis FM, Grout TG, Mohamed SA, Sétamou M, Borgemeister C, Heya HM, Tanga CM, Nderitu PW, Seguni ZS, Materu CL, Ekesi S. (2017). Detection of *Diaphorina citri* Kuwayama (Hemiptera: Liviidae) in Kenya and potential implication for the spread of Huanglongbing disease in East Africa. Biological Invasions. 2017;19:2777-2787.

Ruíz-Rivero O, Garcia-Lor A, Rojas-Panadero B, Franco JC, Khamis FM, Kruger K, Cifuentes D, Bielza P, Tena A, Urbaneja A, Pérez-Hedo M. Insights into the origin of the invasive populations of *Trioza erytreae* in Europe using microsatellite markers and mtDNA barcoding approaches. Scientific Reports. 2021;11(1):18651.

Sjölund MJ, Ouvrard D, Kenyon D, Highet F. Developing an RT-PCR assay for the identification of psyllid species. In Proceedings crop protection in Northern Britain (p. 4). 2016.

Sumner-Kalkun JC, Sjölund MJ, Arnsdorf YM, Carnegie M, Highet F, Ouvrard D, Greenslade AFC, Bell JR, Sigvald R, Kenyon DM. A diagnostic real-time PCR assay for the rapid identification of the tomato-potato psyllid, *Bactericera cockerelli* (Šulc, 1909) and development of a psyllid barcoding database. Plos one. 2020;15(3):e0230741.

Ugwu JA, Ombura FL, Salifu D, Khamis FM. Morphometric and molecular characterization of iroko gall bug, *Phytolyma* species (Hemiptera: Psyllidae) from eastern and western Nigeria. Journal of Research in Forestry, Wildlife and Environment. 2019;11(2):57-68.

Wang Y, Cen Y, Jiang H, Luo X, Deng X, Xia Y. Identification of haplotypes of *Cacopsylla citrisuga* from Yunnan Province based on mitochondria COI sequence. Journal of South China Agricultural University. 2015;36(4):81-86.

Wamonje FO, Zhou N, Bamrah R, Wist T, Prager SM. Detection and identification of a ‘*Candidatus* Liberibacter solanacearum’ species from ash tree infesting psyllids. Phytopathology®. 2022;112(1):76-80.

Zhao Q, Jiang LL, Guo J, Zhang DK, Hu HY. Differences in gall induction of flower-like galls on haloxylon by psyllids (hemiptera: aphalaridae), and the emergence of corresponding parasitoids. Insects. 2021;12(10):861.
